# Organic Carbon Storage in China's Urban Areas

**DOI:** 10.1371/journal.pone.0071975

**Published:** 2013-08-26

**Authors:** Shuqing Zhao, Chao Zhu, Decheng Zhou, Dian Huang, Jeremy Werner

**Affiliations:** 1 College of Urban and Environmental Sciences, and Key Laboratory for Earth Surface Processes of the Ministry of Education, Peking University, Beijing, China; 2 U.S. Geological Survey (USGS) Earth Resources Observation and Science (EROS) Center, Sioux Falls, South Dakota, United States of America; DOE Pacific Northwest National Laboratory, United States of America

## Abstract

China has been experiencing rapid urbanization in parallel with its economic boom over the past three decades. To date, the organic carbon storage in China's urban areas has not been quantified. Here, using data compiled from literature review and statistical yearbooks, we estimated that total carbon storage in China's urban areas was 577±60 Tg C (1 Tg  = 10^12^ g) in 2006. Soil was the largest contributor to total carbon storage (56%), followed by buildings (36%), and vegetation (7%), while carbon storage in humans was relatively small (1%). The carbon density in China's urban areas was 17.1±1.8 kg C m^−2^, about two times the national average of all lands. The most sensitive variable in estimating urban carbon storage was urban area. Examining urban carbon storages over a wide range of spatial extents in China and in the United States, we found a strong linear relationship between total urban carbon storage and total urban area, with a specific urban carbon storage of 16 Tg C for every 1,000 km^2^ urban area. This value might be useful for estimating urban carbon storage at regional to global scales. Our results also showed that the fraction of carbon storage in urban green spaces was still much lower in China relative to western countries, suggesting a great potential to mitigate climate change through urban greening and green spaces management in China.

## Introduction

Urbanization is accelerating worldwide because of rapid population growth and demographic shift from rural to urbanized areas. The proportion of the global population in urban areas rose from 29.4% to 52.1% between 1950 and 2011, and it is expected to increase to 67.2% by 2050 [1]. Although urban areas cover less than 3% of the land surface globally at present [2], [3], their impacts are diverse and extended far beyond city boundaries [4], [5]. The urbanization has caused serious ecological consequences such as the alteration of local to global biogeochemical cycles [6]–[8]. Unlike natural ecosystems which are fueled by solar energy, the maintenance of urban ecosystems relies heavily on fossil fuels. As a result, urban areas account for more than 75% of global anthropogenic carbon dioxide (CO_2_) emissions [8], [9].

On the other hand, urban areas have been found to be a large organic carbon pool. For example, carbon storage in urban and exurban areas of the conterminous United States accounted for 10% of its total land carbon storage in 2000 [10]. The aboveground live biomass carbon stored within the Seattle, WA region was considerably larger than the average US forest carbon stock [11]. The soils in residential turf grass of Baltimore and Denver might accumulate carbon at a rate of about two-fold higher than native ones [12]. There was a substantial amount of carbon stored within aboveground vegetation and soil in Leicester: vegetation carbon density (i.e., carbon storage per unit area) was roughly seven times the average carbon density in the county [13], and soil carbon density was roughly 1.5 times that of regional agricultural land at equivalent soil depths [14]. One important question is to what extent anthropogenic carbon dioxide emissions originated from urban areas can be offset through preserving or increasing carbon storage within urban areas themselves? Unfortunately, the urban carbon balance and its role in global carbon budget remain largely neglected.

As the world's most populous country, China has experienced rapid urbanization in parallel with its economic boom over the past three decades. From 1975 to 2006, the number of cities grew from 193 to 656 [15], and the proportion of urban population increased from 17.4% to 43.9% [16]. Increased economic growth and urbanization means greater energy consumption and therefore more CO_2_ emissions. It was reported that fossil-fuel carbon emissions in China increased nearly four-fold from 1980 to 2006, making the country the world's largest contributor to CO_2_ emissions for the first time [17]. By 2050, China's population is projected to be 1.3 billion with 77.3% living in cities [1]. A good understanding of China's urban carbon stock and budget is critical to mitigating climate change not just for China, but the entire world. However, to date there are no such data available.

In this study, for the first time, we estimated the organic carbon storage (including both natural and anthropogenic pools) in China's urban areas at national and regional scales for 2006 using data compiled from literature review and statistical yearbooks. We quantified the uncertainty of the estimates and performed a sensitivity analysis. A horizontal comparison with the carbon storage in the urban areas of the United States was also made to shed light on the differences in carbon storage between these two major countries and the likely controlling factors.

## Materials and Methods

### Study area

There are five levels of administrative units of the cities in China: (1) provincial-level cities, (2) deputy-provincial cities, (3) provincial capitals, (4) prefecture-level cities, and (5) county-level cities [18]. We made a preliminary estimation of carbon storage in 287 cities above county-level of China in a previous study [19]. In this study we considered the cities of mainland China at all levels, and the number of cities was 656 with a total area of 3.37×10^4^ km^2^ in 2006, which accounted for 0.35% of the country's total land area (9.60×10^6^ km^2^). We estimated the organic carbon storage in these 656 cities for the year 2006 at national and regional scales with more extensive literature review. The major regions of China consist of North China, Northeast China, East China, Central-south China, Southwest China and Northwest China. The coverage of each of the regions is listed in Table S1.

### Data and Methods

Organic carbon in urban area is stored not only in natural pools such as vegetation and soils but also in anthropogenic ones including buildings and humans. We estimated carbon storage of these four pools in China's urban areas largely following the method of Churkina *et al*. [10], but added a procedure to estimate uncertainty and performed a sensitivity analysis.

### Carbon storage in vegetation and soils

Vegetation in urban areas mainly consists of urban forest and urban grassland. In this study, we considered urban forest and grassland as one pool (i.e., urban green spaces) because the available data in China's urban areas do not make a distinction between them. We estimated vegetation carbon stock (*C_green_*) in urban area with the following equation:
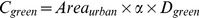
(1)Where *Area_urban_* represents the urban area (m^2^), α represents the percentage of green space in urban area (%), and *D_green_* represents the vegetation carbon density of green spaces (kg C m^–2^).

Carbon storage in soils (*C_soil_*) was calculated as a sum of carbon stored in soils beneath impervious surfaces and green spaces, assuming that all urban soils were covered by impervious surfaces and green spaces:
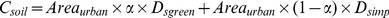
(2)where *D_sgreen_* and *D_simp_* represent the carbon density of soils beneath green space and impervious surfaces (kg C m^–2^), respectively. Since most soil carbon surveys reach to 100 cm in depth wherever possible [20], we estimated carbon storage in urban soils to the same depth to facilitate comparison with other studies.

The information on urban area and percentage of green space in urban areas was obtained from China Urban Construction Statistical Yearbook [21]. The vegetation carbon densities in urban green spaces and carbon densities of soils beneath green spaces and impervious surfaces were compiled from literature review by provinces (municipalities or autonomous regions). For provinces where carbon density data were not available, corresponding carbon density values in the same region were used as a substitute to estimate their carbon storage (Table S1).

### Carbon storage in humans

Humans and pets can also store organic carbon. We focused only on carbon storage in humans because carbon storage in pets was equivalent to less than 1% of humans [22]. Carbon storage in humans (*C_hum_*) was calculated as follows:
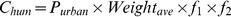
(3)where *P_urban_* represents the population in urban area, Weight_ave_ represents the average human body weight (60 kg), f_1_ and f_2_ is the fraction of dry organic matter in human body (0.3) [22] and the fraction of carbon in dry organic matter (0.5) [23], respectively. The population data in urban areas were obtained from China Population and Employment Statistics Yearbook [24].

### Carbon storage in buildings

In a building system, organic carbon is mainly stored in constructive materials, decorated materials, furniture, books, and foods, etc. We focused on carbon storage in constructive materials (*C_constru_*) and furniture (*C_furn_*) assuming carbon stored in other pools was relatively smaller [10]. We distinguished commercial buildings from residential buildings since wood use in the two types are different. First, we calculated the amount of wood used in constructive materials and furniture, and then we transformed the figure to carbon storage using f_2_:
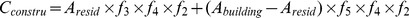
(4)

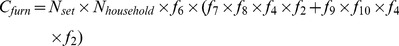
(5)where *A_resid_* and *A_building_* represent the floor area of residential buildings and total buildings in urban areas (10^4^ m^2^), respectively; *N_set_* represents the number of composite furniture owned by per household (set); *N_household_* represents the number of household in urban areas (household); f_3_ and f_5_ represent the wood use per unit of residential buildings floor area and commercial buildings floor area (0.045 and 0.055 m^3^·m^−2^) [25], respectively; f_4_ represents the average bulk density of wood (0.4 t·m^−3^) [26]; f_6_ represents the number of pieces per set of composite furniture (10–30, with an average number of 20, assumed based on the communications with furniture dealers); f_7_ and f_9_ represent the fraction of wood furniture and steel furniture in total composite furniture (0.8 and 0.2) [27], respectively; while f_8_ and f_10_ represent the wood use per unit of wood furniture and steel furniture (0.067 and 0.026 m^3^·piece^−1^) [28], respectively. The data on the floor area of residential buildings and total buildings, and the number of composite furniture owned by per household in urban areas were compiled from China Statistical Yearbook [16], China Statistical Yearbook for Regional Economy [29]. The number of household in urban areas was compiled from China Population and Employment Statistics Yearbook [24].

### Estimation of Uncertainty

Most of the variables used in the calculation of carbon storage in various pools were poorly studied in urban areas. The estimates of these variables were derived from a few isolated studies or from census reports, and their uncertainty was difficult to quantify but expected to be large. In this study, we assumed the uncertainty of all variables except the soil carbon contents follows the normal distribution with mean of 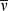 and variance of 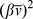 where β is a constant coefficient indicating the level of uncertainty was proportional to 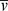. Soil carbon content usually follows a log-normal distribution [30], which was used to represent the distribution of soil carbon content under the green cover and impervious surfaces in this study as follows:
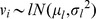
With



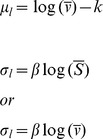
where *v_i_* is the estimate of a given soil carbon content variable, 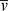 is the estimated mean of the variable, 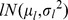 is a log-normal distribution, β is the same constant variation coefficient as above, 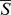 is the given standard deviation of 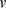, and k is a small constant used to transform the mean of the distribution found through Monte Carlo simulation and numerical optimization. The standard deviation of soil carbon content under impervious surfaces was estimated from a national database (Table S1), which was directly used in estimating carbon storage uncertainty as a value for 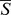. For other variables, a value of 0.15 for β was used to estimate variability using equation 6, as available information was not enough to estimate their standard deviation. Using these methods, a total of 10,000 Monte Carlo simulations per province were executed for the input variables to find total carbon values and uncertainty using equations 1–5. The values for all variables in each of the 10,000 calculations were randomly and independently drawn from the normal or log-normal distributions using a Monte Carlo procedure in R [31].

## Results

**Figure 1:**
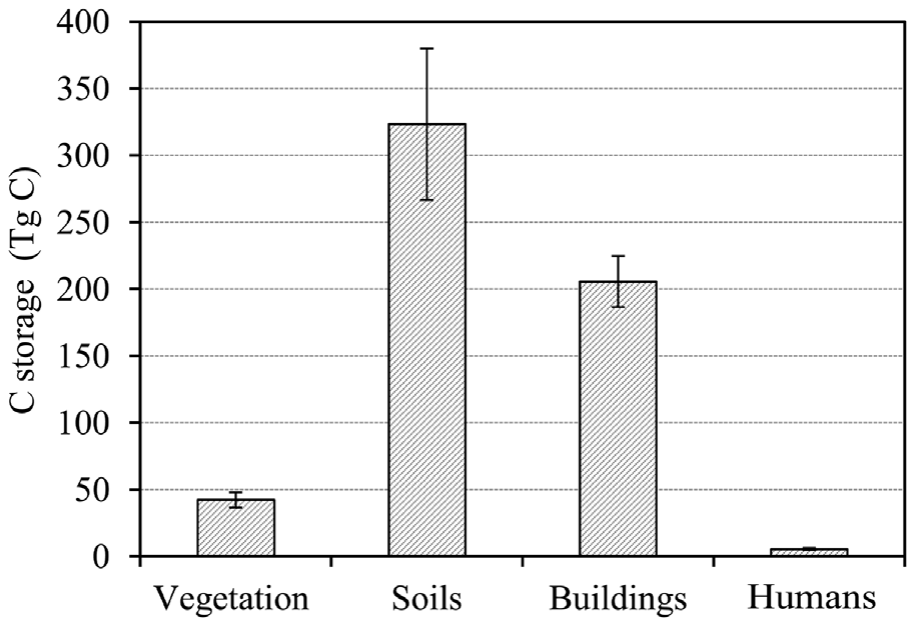
Carbon storage in four major pools in China's urban areas in 2006 at 95% confidence intervals (i.e., the mean±1.96× standard error).

The total carbon storage in China's urban areas was 577±60 Tg C (95% confidence intervals, hereafter) in 2006 with a total area of 3.37×10^4^ km^2^ (Table 1). The average carbon density in urban areas was 17.1±1.8 kg C m^−2^. Soil was the largest carbon pool accounting for 56.1% of the total carbon storage, followed by buildings (35.6%) and vegetation (7.3%), while humans pool was relatively small (1%) (Figure 1 and Table 2).

**Table 1:** Carbon storage and density in urban areas of China in 2006 at national and regional scales at 95% confidence intervals.

Region	Area (km^2^)	Total (Tg C)	C Density (kg C m^−2^)
North China	4775	70.2±12.8	14.7±2.7
Northeast China	4340	88.8± 38.4	20.5± 8.9
East China	10756	181.4±23.8	16.9± 2.2
Central-south China	8691	147.1±32.4	16.9±3.7
Southwest China	2929	61.7±18.2	21.1±6.2
Northwest China	2205	27.8±5.3	12.6±2.4
China	33697	577.0±60.1	17.1±1.8

**Table 2:** The proportion of total carbon in urban areas of China stored in four major carbon pools at regional scales and for the country as a whole for the year 2006.

Region	Vegetation (%)	Soil (%)	Humans (%)	Buildings (%)
North China	4.9	55.3	0.8	39.0
Northeast China	7.1	69.8	0.7	22.4
East China	10.3	49.9	1.0	38.8
Central-south China	6.9	55.7	1.1	36.3
Southwest China	4.8	57.5	1.0	36.7
Northwest China	3.0	53.1	1.1	42.8
China	7.3	56.1	1.0	35.6

At the regional scale, cities in the East and Central-south regions stored the largest amount of organic carbon, with an estimate of 181±24 Tg C and 147±32 Tg C, accounting for 31.4% and 25.5% of total national urban carbon storage, respectively. The Northeast and North regions also played a significant role in organic carbon storage of China's urban areas, shared 15.4% and 12.2% of the total, respectively, while the urban areas in Southwest and Northwest regions had the lowest carbon storage (Table 1).

**Figure 2:**
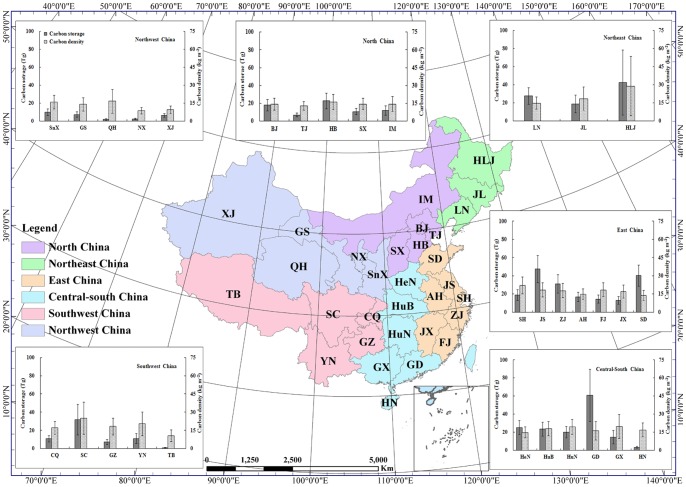
Carbon storage (Tg) and density (kg C m^−2^) in the provinces (municipalities or autonomous regions) in China's urban areas in 2006 (except Taiwan, Hong Kong and Macao). Carbon storage is the sum of four pools: vegetation, soils, humans and buildings. The confidence intervals are the same as shown in Figure 1.

Carbon storage in urban areas varied significantly among different provinces (Figure 2), ranging from 0.8±0.4 Tg C in Tibet to 60.7±29 Tg C in Guangdong. In general, urban areas in the eastern provinces stored more organic carbon than western provinces. In most provinces (23 of 31), the carbon storage was below 25 Tg, whereas Guangdong accounted for 10.5% of total carbon storage in China's urban areas.

The contribution of each pool to total carbon storage in each region was similar to the national pattern (Table 2). However, there were some differences in magnitude. The proportion of carbon storage in vegetation was highest in the East (10.3%) and lowest in the Northwest (3.0%). The fraction of carbon stored in soil in the Northeast (69.8%) was much higher than the national average (56.1%).

The average carbon density in urban areas increased from 12.6±2.4 kg C m^−2^ in Northwest to 21.2±6.2 kg C m^−2^ in Southwest (Table 1). The largest carbon density was found in Helongjiang at 28.9±24.6 kg C m^−2^, and the lowest was found in Ningxia with 8.6±2.7 kg C m^−2^, with most provinces (26 of 31) having carbon density between 12 kg C m^−2^ and 22 kg C m^−2^ (Figure 2).

**Figure 3:**
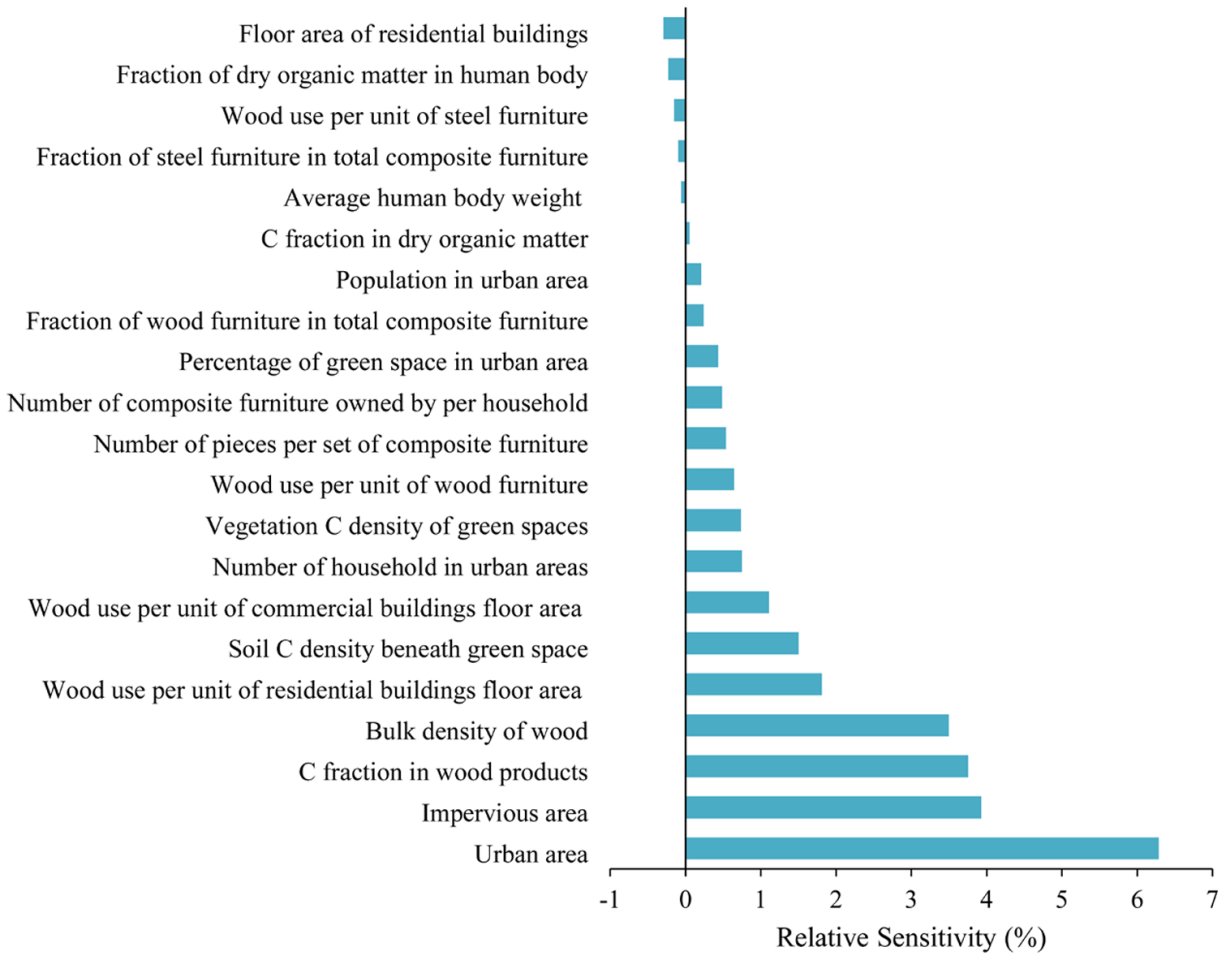
Sensitivity of total urban carbon storage to a 10% increase in the each of the input variables.

Figure 3 shows the relative response or sensitivity of the total urban carbon storage on 10 percent increase of the each of the input variables. The relative sensitivities of all variables used in estimating urban carbon storage in China were smaller than 10 percent, indicating the uncertainties of these variables would be contracted in the estimated carbon storage. The most sensitive variable was urban area with a sensitivity of 6.3 percent, followed by the second group of variables (i.e., impervious area, carbon fraction in wood products, and bulk density of wood) with sensitivity ranging from 3.5 to 3.9 percent. The third group of variables with a sensitivity varying between 1.1 to 1.8 percent included wood use per unit of residential buildings floor area and commercial buildings floor area, and soil C density beneath green space. The sensitivities of the rest of the variables were all smaller than 1 percent with five of the 21 variables being negative. The sensitivity analysis clearly suggested that reducing the uncertainty in the estimated urban area, impervious area or green space, carbon fraction in wood products, and bulk density of wood might be the most cost-effective in reducing the total uncertainty in the estimated urban carbon storage in China.

## Discussion

Our estimate of organic carbon stored in China's urban areas was 577±60 Tg C or ranged from 517 to 637 Tg C in 2006, while the total carbon storage in China's terrestrial ecosystems was estimated to be 77.4 Pg C (1 Pg  = 10^15^ g) [32]–[34]. This suggests that urban areas accounted for 0.74% of the country's terrestrial ecosystems carbon storage, yet they represent only 0.35% of its land area. In other words, the carbon density in urban areas was 2.1 times the national average carbon density.

At both the national and regional scales, soils accounted for most of the total urban carbon storage, followed by buildings, vegetation, and humans. However, the partitioning varied regionally. Urban soils in the Northeast had the highest proportion of carbon storage across China, signifying the relatively larger impact of soil storage in this region than others. This is consistent with the finding from natural ecosystems that soils under cold climate generally have higher carbon densities [35], [36]. The highest proportion of vegetation carbon storage was observed in the East where both urban green space coverage and vegetation carbon density were relatively and simultaneously high, and the lowest proportion was found in the Northwest region where both urban green space coverage and vegetation carbon density were the lowest. Regional differences in vegetation carbon storage can be attributed to climate, soil, and habitat conditions as well as urbanization process and history [33].

**Figure 4:**
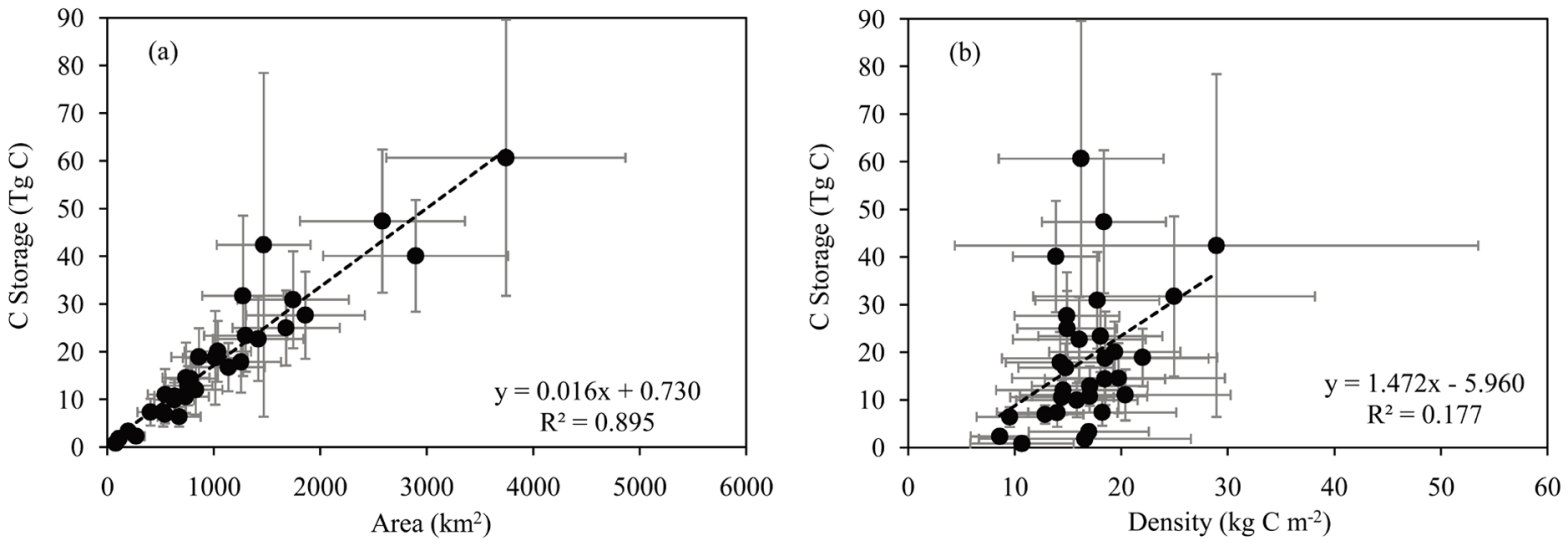
Relationship between urban carbon storage and urban area (a) or urban carbon density (b) at the provincial level. The confidence intervals are the same as shown in Figure 1.

Urban carbon storage strongly depends on urban land area and less on carbon density (Figure 4). The regions or provinces with larger urban land areas usually stored more carbon (Table 1 and Figure 4a). East and Central-south regions accounted for 57.7% of total urban land areas and stored 56.9% of total carbon. Guangdong province was the largest in urban areas among all provinces (11.1% of national urban areas), contributing most to the national urban carbon storage (10.5%). In contrast, Northwest region only stored 4.8% of total carbon with 6.5% of total urban area. Tibet, the least urbanized province in China, had the lowest urban carbon storage. These results suggest that urban carbon storage increase in China is more likely to be caused by urban expansion. Considering the rapid urbanization rate in China, the role of urban areas in carbon cycle of terrestrial ecosystems will become more significant. Urban carbon storage is also related to its carbon density, but this relationship is much weaker than that between storage and area (Figures 4a and b).

**Figure 5:**
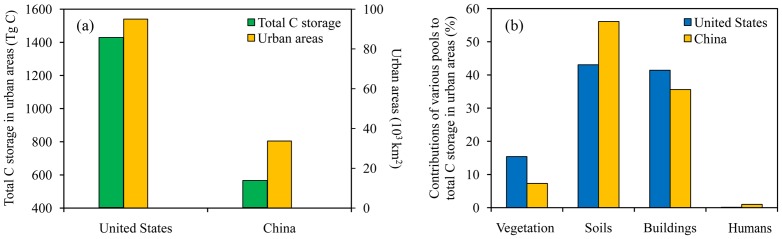
Comparisons of the total urban carbon storage and urban areas (a), and the fractions of four major carbon pools (b) between China and the Conterminous United States.

Similarity and differences of urban carbon storage and its partitioning among various carbon pools between China and the United States might provide insights for future efforts in estimating urban carbon storage in other countries. The urban carbon storage in China estimated in this study is about 38% of that in the United States. This suggests that urban carbon densities between these two countries were comparable as the urban area in China was 36% of that in the United States (Figure 5a). The partitioning of total carbon storage in China was different from that in the United States (Figure 5b). Obviously, the contributions of vegetation pool and buildings to total urban organic carbon storage in the United States were higher than those in China. This can probably be explained by the following two factors. First, urban greening has been practiced for many years in the United States. In contrast, urban greening has just gained increasing attention in recent years in China [37]. The coverage of green spaces in the urban areas of the United States was 73% overall with 27% covered by trees and 46%, with large uncertainty, by other green covers [10], [38], [39]. On the other hand, the total green space (including both forests and grasses) in China's urban areas was only 31% in 2006 [21], less than half of its counterpart in the United States. Second, in addition to the difference in the number or area of buildings, differences in other factors such as wood use in constructions and furniture contributed to the disparity in carbon storage in buildings between these two countries. For example, wood use in construction materials was 18–22 kg m^–2^ in China, less than half of that (40–130 kg m^–2^) in the United State [10], [28]. Our estimate of organic carbon storage in buildings in China's urban areas was 0.34×10^6^ g C per capita, which was less than thos× of industrialized countries (2.1×10^6^ g C per capita) and higher than those of less industrialized countries (0.15×10^6^ g C per capita) [40]. In contrast, the organic carbon stored in buildings in the United States amounted to 3.06×10^6^ g C per capita [10].

In this study, we made a preliminary estimation on organic carbon storage in China's urban areas based on the data compiled from literature review and statistical yearbooks. However, the result remains uncertain mainly due to uncertainty from the input data (both in statistical data and measured data). For instance, these data did not have a good measure on the quality of green space (i.e., the type of green space, and carbon density in vegetation and soils for each type). Most statistical data in China do not distinguish urban forest from urban grassland, although carbon density in urban forest might be very different from that in urban grassland. For example, previous studies documented that aboveground carbon density of urban forest in the United States was 8.6 kg C m^−2^, about 170 times as large as its counterpart in urban grassland [10], [41]. Wen *et al*. [42] found that biomass of urban trees and urban grassland in the city of Taizhou, East China, was 2.48 and 0.44 kg C m^−2^, respectively. Future differentiation of urban forests from grasslands using remote sensing or inventory approaches may reduce the uncertainty.

Since population statistics in China mainly based on *hukou* (household registration system) population data which does not include floating population in cities [43], the numbers are often underestimated. However, the impact on estimating total urban carbon storage is trivial as the carbon storage in humans only accounts for 1% of the total urban carbon storage. This observation applies to cities in both China and the United States (Figure 5b), and probably applies to other cities in the world as well. Carbon storage in humans accounts for about 0.1% of the total urban carbon storage in the United States. Because of the very small contribution of humans to total urban carbon storage in general, we advocate that this term be dropped from future efforts in estimating urban carbon storage.

The slope of the strong linear relationship in Figure 4a indicates that on average the total carbon storage is 16 Tg C for every 1,000 km^2^ urban area, which is very close to 15.9 Tg C per 1,000 km^2^ urban area found in the United States [10]. This specific urban storage capacity might be extrapolated to other regions of the world as the relationship covered a wide range of regions from arid to moist and from developing to developed countries. Using this specific urban carbon storage capacity and the minimum and maximum estimates of global urban areas (276,000–3,524,000 km^2^) [3], we calculated that the global total urban carbon storage would be within the range of 4.4 to 56.4 Pg C. The large bound was caused by the range of estimates of the global urban area. Sensitivity analysis results also suggested that urban carbon storage in China was most sensitive to total urban area (Figure 3). Therefore, reducing uncertainty in total urban area might be the most effective way to decrease uncertainty in urban carbon storage estimates.

Urban greening could be a practical approach to contribute to achieve policy targets of mitigating climate change compared with other direct greenhouse gas (GHG) emission reduction strategies [13], [44]. Our results showed that the fraction of carbon storage in urban green spaces was still much lower in China than in western countries. Therefore increasing carbon storage in urban green spaces in China has great potential to mitigate carbon emissions in urban areas and also contribute to the target of reducing China's GHG emissions per unit of gross domestic product (GDP) by 40 to 45% in 2020 as compared to that of 2005 [45]. This highlights the importance of urban greening and green spaces management in China.

## Conclusions

This study estimated for the first time that the organic carbon storage in the urban systems of China ranged from 517 to 637 Tg C (95% confidence interval) in soils, vegetation, buildings, and humans in the year 2006. Soil accounted for 56% of the total carbon storage, followed by buildings (36%), and vegetation (7%). The weight of humans was only about 1% of the total organic carbon storage and therefore could be ignored in the study of carbon storage in urban systems.

Several lines of evidence suggest the increasing importance of urban systems in regional and national carbon study and management. First, on a per unit area basis, the urban systems of China stored about two times the national average of all lands, which has largely been ignored in previous studies. Second, China's total urban area will likely continue to increase due to continued economic development. Third, there is still room for carbon storage enhancement in the urban green spaces of China compared with its equivalent in the United States.

Some major future research directions have been identified through uncertainty and sensitivity analysis. Uncertainty analysis indicates that urban soil carbon storage had the largest uncertainty, followed by buildings and vegetation. It is therefore important to systematically collect field samples to measure the spatial variability of soil carbon density under various cover types across geographic regions. In addition, sensitivity analysis indicates carbon storage estimate was most sensitive to the accuracy of the estimates of urban area and impervious area (or the area of green space). The quality of green space, represented by the carbon density in vegetation and soils, was also an important factor for reducing uncertainty in estimating carbon storage in China's urban systems.

## Supporting Information

Table S1 **Carbon densities of vegetation and soils in urban areas of China's 31 provinces (municipalities or autonomous regions).**
(DOCX)
