# Supplementary material for: Organic Carbon Storage in China's Urban Areas
Source: PLoS One. 2013 Aug 26;8(8):e71975. doi: 10.1371/journal.pone.0071975 (PMC3753297; doi:10.1371/journal.pone.0071975)
Supplement: Table S1 — Carbon densities of vegetation and soils in urban areas of China's 31 provinces (municipalities or autonomous regions). (DOCX) [file pone.0071975.s001.docx]

**SUPPLEMENTAL INFORMATION FOR**

Organic Carbon Storage in China’s Urban Areas

Shuqing Zhao^1,*^, Chao Zhu^1^, Decheng Zhou^1^, Dian Huang^1^, Jeremy Werner^2^

^1^ College of Urban and Environmental Sciences, and Key Laboratory for Earth Surface Processes of the Ministry of Education, Peking University, Beijing, China

^2^ U.S. Geological Survey (USGS) Earth Resources Observation and Science (EROS) Center, Sioux Falls, South Dakota, USA

*Correspondence to: S. Q. Zhao (sqzhao@urban.pku.edu.cn); phone/fax: +86-10-6276 7707

Table S1. Carbon densities of vegetation and soils in urban areas of China's 31 provinces (municipalities or autonomous regions)

| Regions | Provinces | Carbon density (kg C m^-2^) | | | | |
| --- | --- | --- | --- | --- | --- | --- |
|  |  |  |  | Soils |  |  |
|  |  | Vegetation* | Reference | Impervious ^†^ | Green spaces^‡^ | Reference |
| North China | Beijing | 2.28 | 46 | 8.9 ± 8.1 | 7.26 | 46 |
|  | Tianjin |  |  | 6.4 ± 2.9 |  |  |
|  | Hebei |  |  | 9.6 ± 9.3 |  |  |
|  | Shanxi |  |  | 6.6 ± 7.5 |  |  |
|  | Inner Mongolia |  |  | 9.4 ± 9.8 |  |  |
|  |  |  |  |  |  |  |
| Northeast | Liaoning | 4.95 ^Â§^ | 47, 48 | 8.4 ± 5.6 | 8.63 ± 0.83 | 56 |
| China | Jilin |  |  | 13.3 ± 14.6 | 10.68 ± 0.43 ^**^ |  |
|  | Heilongjiang |  |  | 23.4 ± 38.8 | 12.73 ± 0.20 | 57 |
|  |  |  |  |  |  |  |
| East China | Shanghai | 4.25-5.57 | 49 | 11.7 ± 5.8 | 9.29 ± 1.05 | 58, 59, 60 |
|  | Jiangsu | 5.86 | 50 | 7.9 ± 4 | 11.58 ± 3.40 | 61, 62 |
|  | Zhejiang | 4.63 ± 0.97 | 51 | 9.4 ± 5.9 | 10.13 ± 1.16^**^ | 63 |
|  | Anhui | 5.09 ± 0.32 ^Â¶^ |  | 7.5 ± 4.2 | 5.75-14.27 | 64 |
|  | Fujian | 5.09 ± 0.32 ^Â¶^ |  | 9.4 ± 5 | 9.38 ± 0.79 | 65, 66 |
|  | Jiangxi | 5.09 ± 0.32 ^Â¶^ |  | 8.8 ± 5.4 | 9.38 ± 0.79 ^**^ |  |
|  | Shandong | 5.09 ± 0.32 ^Â¶^ |  | 6.3 ± 5.4 | 6.09 ± 0.68 ^**^ | 67 |
|  |  |  |  |  |  |  |
| Central-south | Hunan | 4.59 ^Â§^ | 52 | 6.7 ± 5 | 9.12 ± 0.66 | 68, 69 |
| China | Guangdong | 3.30 | 53 | 9.4 ± 6.2 | 9.32 ± 0.29 | 53, 70,71,72,73,74 |
|  |  |  |  |  |  |  |
|  | Henan | 3.94 ^Â¶^ |  | 9.9 ± 5.8 | 6.99-11.02 | 75 |
|  | Hubei | 3.94 ^Â¶^ |  | 9.6 ± 18.4 | 6.65-18.46 | 76 |
|  | Guangxi | 3.94 ^Â¶^ |  | 12.3 ± 20.7 | 7.97-8.67 | 77 |
|  | Hainan | 3.94 ^Â¶^ |  | 10.3 ± 5.2 | 9.34-9.66 | 78 |
|  |  |  |  |  |  |  |
| Southwest | Chongqing |  |  | 7.8 ± 5.8 | 8.11 ± 2.67 | 79 |
| China | Sichuan |  |  | 16 ± 21.3 | 8.34-9.72 | 80 |
|  | Guizhou |  |  | 12 ± 6.3 | 11.44 ± 0.66 | 81 |
|  | Yunnan | 3.86 ^Â§^ | 54 | 13.5 ± 13.5 | 6.54-8.44 | 82 |
|  | Tibet |  |  | 6.9 ± 9.9 | 9.05 ± 0.69 ^Â¶^ |  |
|  |  |  |  |  |  |  |
| Northwest | Shaanxi | 1.52 ± 0.04 ^\|\|^ | 55 | 7.4 ± 8.6 | 10.51 ± 0.59 | 83 |
| China | Gansu |  |  | 7.6 ± 9.6 | 6.26 ± 0.20 | 84 |
|  | Ningxia |  |  | 13.2 ± 16 | 4.08-5.36 | 85 |
|  | Qinghai |  |  | 5 ± 4.3 | 4.08-5.36^**^ |  |
|  | Xinjiang |  |  | 4.6 ± 6.2 | 4.75 ± 0.89 | 86, 87 |

* For regions where there is only one vegetation carbon density available, we regarded it as the average carbon density of vegetation in the whole region; for regions which have more than one data, we used measured data to estimate carbon storage in urban vegetation for corresponding provinces, and used mean value of measured data in the same region to estimate carbon storage in urban vegetation for provinces where there are no data available.

^†^ Carbon densities of soil beneath impervious surfaces were not available in literature therefore we estimated impervious surfaces carbon storage based on average soil carbon density in China’s 31 provinces, assuming that the two densities were equal since carbon density of soil is stable once covered by impervious surfaces [59]. The average soil carbon densities and their standard deviations for the 31 provinces were provided by Dr. DS Yu from Institute of Soil Science, Chinese Academy of Sciences, which were the original data of Yu et al. [88].

^‡^ Soil organic carbon (SOC) densities beneath green spaces for 100 cm depth were needed in this study, but we found many publications did not report these data. Instead, they reported soil organic matter (SOM) or SOC contents or SOC densities beneath green spaces less than 100 cm. Therefore, in order to include as many studies as possible, we calculated SOC densities beneath green spaces for 100 cm depth from above data using methods developed by Yang et al. [34].

^Â§^In some cases carbon densities were not given directly, we calculated them from available data in the corresponding literature.

^Â¶^ Mean value calculated from data of other provinces in the same region.

^||^ Data in this region were not available; therefore we used mean value of the whole China instead.

^**^ There were no measured data in these provinces; hence, we used data with the same soil type as a substitute.

**References**

1. Wang DS (2009) Studies on net carbon reserves in Beijing urban landscape green based on biomass measurement. PhD Dissertation. Beijing: Beijing Forestry University. 141 p.
2. Liu CF, He XY, Chen W, Zhao GL (2006) Analysis of environmental benefits of vegetation in Shenyang built- up areas. Journal of Liaoning Forestry Science & Technology: 1-3.
3. Liu CF, He XY, Chen W, Zhao GL, Li L, Xu WD (2008) Ecological benefit evaluation of urban forests in Shenyang City based on QuickBird image and CITYgreen model. Chinese Journal of Applied Ecology 19: 1865-1870.
4. Xu F, Liu WH, Ren WL, Zhong QC, Zhang GL, Wang KY (2010) Effects of community structure on carbon fixation of urban forests in Shanghai, China. Chinese Journal of Applied Ecology 29: 439- 447.
5. Peng L, Chen S, Liu Y, Wang J (2008) Application of CITYgreen model in benefit assessment of Nanjing urban green space in carbon fixation and runoff reduction. Frontiers of forestry in China 3: 177-182.
6. Wen JS (2010) Effects of urbanization on carbon storage and sequestration in the built- up area. M.S. Thesis. Hangzhou: Zhejiang University. 65 p.
7. Gao SC (2010) Nutrient cycling and carbon balance of urban forest ecosystem in Changsha. PhD Dissertation. Changsha: Central South University of Forestry &Technology. 133p.
8. Guan DS, Chen YJ (2003) Roles of urban vegetation on balance of carbon and oxygen in Guangzhou. Journal of Environmental Sciences 15: 155-159.
9. Liu JT, Zhao LS (2008) Quantitative analysis on partial ecological benefits and related tending cost of Jinbi Park, Kunming. Journal of Northwest Forestry University 23: 214- 217.
10. Yu G, Li X, Wang Q, Li S (2010) Carbon storage and its spatial pattern of terrestrial ecosystem in China. Journal of Resources and Ecology 1: 97-109.
11. Duan YQ, Wei ZY, Han CL, Kong LS, Wang QB (2008) Contents of organic carbon urban soil in different land use type areas, Northeast China. Journal of Shenyang Agricultural University 39: 324- 326.
12. Gao YJ (2002) Studies on soil of Harbin urban vegetation. M.S. Thesis. Harbin: Northeast Forestry University. 37 p.
13. Shi LJ, Zheng LB, Mei XY, Yu LZ, Jia ZC (2010) Characteristics of soil organic carbon and total nitrogen under different land use types in Shanghai. Chinese Journal of Applied Ecology 21: 2279- 2287.
14. Xu NZ, Zhang TL, Wang XX, Liu HY (2011a) Analysis on evolution trend of soil organic carbon stock during the course of urbanization. Chinese Journal of Soil Science 42: 659- 663.
15. Fang HL, Chen L, Huang YZ, Zhang Q, Xi YW, Zhao XY (2007) Current situation and strategy for the soil quality of newly-established green belts in Shanghai. Scientia Silvae Sinicae 43: 89-94.
16. He Y, Zhang GL (2006) Concentration and sources of organic carbon and black carbon of urban soils in Nanjing. Acta Pedologica Sinica 43: 177- 182.
17. Wang XZ (2006) The character and distribution of the greenbelt soil of Nanjing Park. M.S. Thesis. Nanjing: Nanjing Forestry University. 61 p.
18. Xu NZ, Zhang TL, Wang XX, Liu HY (2011b) Soil organic carbon storage changes in Yangtze Delta region, China. Environmental Earth Sciences 63: 1021-1028.
19. Wu SS (2010) Carbon storage of the different urban communities in Huancheng Park in Hefei. M.S. Thesis. Hefei: Anhui Agricultural University. 50 p.
20. Dong Y, Tong C, Yang HY, Liu BG, Yan ZP (2007) Soil organic carbon content in urban natural and man manipulated green-lands of Fuzhou city. Journal of Hangzhou Normal University (Natural Science Edition) 6: 440- 444.
21. Zeng HD, Du ZX, Yang YS, Li XB, Zhang YC, Yang ZF (2010) Effects of land cover change on soil organic carbon and light fraction organic carbon at river banks of Fuzhou urban area. Chinese Journal of Applied Ecology 21: 701- 706.
22. Yu FZ, LiB J (2006) Soil nutrient status of park greenery plot in Xuzhou city zone. Ecologic Science 25: 454-458.
23. Guo JP, Wu FC, Deng YH, Li B (2008) Study on reconstruction and properties of the afforested soil in the urban residential area-Taken the Changtangshan residential district as an example. Yunnan Geographic Environment Research 20: 33- 36.
24. Gao SC, Tian DL, Yan WD, Fang X, Xiang WH, Liang XC (2010) Characteristics of soil physicochemical property and its carbon storage in urban forest plantation of Changsha city. Journal of Central South University of Forestry & Technology 30: 16-22.
25. Li XM (2008) The research on carbon circulation of forest ecosystem in Guangzhou City. M.S. Thesis. Changsha: Central South University of Forestry & Technology. 81 p.
26. Zhuo W S, Tang JF, Guan DS (2007) A study on the soil properties of urban green space in Guangzhou and the impact of human activities on them. Acta Scientiarum Naturalium Universitatis Sunyatseni 46: 32-35.
27. Zhu C, Xiong YM, He MM, Feng YM (2010) Soil physico-chemical properties and their correlations in a greenbelt in Guangzhou. Ecology and Environmental Sciences 19: 1868- 1871.
28. Shi ZJ, LuY, Zhang DM (2008) Modeling of quantitative carbon-nitrogen relationship in urban soils based on different classification methods. Soils 40:561- 565.
29. Hu WJ (2008) Studies on soil physical- chemical properties and enzymes activities for urban parks and roads green-land in Shenzhen. M.S. Thesis. Wuhan: Huazhong Agricultural University. 86 p.
30. Sun Y, Ma J, Li C (2010) Content and densities of soil organic carbon in urban soil in different function districts of Kaifeng. Journal of Geographical Sciences 20: 148-156.
31. Wang P, Hu HH, Ding ZQ (2009) Physic-chemistry properties of soil from urban green space in Wuhan city. Hubei Agricultural Sciences 48: 78- 80.
32. Liu Y (2008) Research on the physiological characteristics of four shrubs species and the property of soil in Nanning. M.S. Thesis. Nanning: Guangxi University. 60 p.
33. Fan HY (2010) Analysis on ecogeographical characteristics of soil animal groups of tropical urban greenbelt in Haikou. M.S. Thesis. Haikou: Hainan Normal University. 57 p.
34. Bao B, Ding WQ, Wu D (2008) Quality of landscape soil in urban area of Chongqing city. Environmental Science & Technology 31: 51- 52.
35. Chen X, Lang CY (2011) Study of soil physicochemistry properties of greenbelt zones of Chengdu. Guangdong Trace Elements Science 18: 52- 57.
36. Ning C, Yan WD, Ye SJ, Sun JH, Liu LD, Yang CH, Liu JD (2011) Biomass and productivity of Betula luminifera and Italian poplar mixed-forests ecosystem in karst city. Journal of Central South University of Forestry & Technology 31: 161-166.
37. Hu H R, Li RK, Liu J (2011) Study on phy-chemical properties of greened soil of residential areas in Kunming. Journal of Anhui Agricultural Sciences 39: 2129- 2131.
38. Sun XF, Xu TT, Wang M, Liu S, Shen XF (2011) Concentration determination and contamination evaluation for heavy metals in soil of three parks inside wall of Xi’an city. Urban Environment & Urban Ecology 24: 1- 4.
39. Kang LF, Li FR, Zhang AS, Tan JA, Yang FW, Hei WL, Liu JB (2006) Effects of traffic pollution on urban soils and plants. Environmental Science 27: 556- 560.
40. Cao B, GaoLF, SuRH, Song LH (2009) Investigation and analysis of soil fertility of urban greenbelts in Yinchuan. Journal of Agricultural Sciences 30: 5- 9.
41. Chai N, Qiu WC, Yin LK, Luo QY, Zhang Z (2008) Comprehensive valuation on soil nutrient of Karamay. Journal of Xinjiang Agricultural University 31: 51- 55.
42. Liu YY, Liu M (2007) Study on the properties and pollution of urban soils in Urumqi. Arid Zone Research 24: 66- 69.
43. Yu DS, Shi XZ, Wang HJ, Sun WX, Chen JM, Liu QH, Zhao YC (2007) Regional patterns of soil organic carbon stocks in China. Journal of Environmental Management 85: 680-689.
